# In situ serial crystallography for rapid de novo membrane protein structure determination

**DOI:** 10.1038/s42003-018-0123-6

**Published:** 2018-08-27

**Authors:** Chia-Ying Huang, Vincent Olieric, Nicole Howe, Rangana Warshamanage, Tobias Weinert, Ezequiel Panepucci, Lutz Vogeley, Shibom Basu, Kay Diederichs, Martin Caffrey, Meitian Wang

**Affiliations:** 1 Swiss Light Source, Paul Scherrer Institute, CH-5232 Villigen, Switzerland; 2 Membrane Structural and Functional Biology (MS&FB) Group, School of Medicine and School of Biochemistry and Immunology, Trinity College Dublin, Dublin 2, D02 R590 Ireland; 3 Fachbereich Biologie, Universität Konstanz, M647, D-78457 Konstanz, Germany

## Abstract

De novo membrane protein structure determination is often limited by the availability of large crystals and the difficulties in obtaining accurate diffraction data for experimental phasing. Here we present a method that combines in situ serial crystallography with de novo phasing for fast, efficient membrane protein structure determination. The method enables systematic diffraction screening and rapid data collection from hundreds of microcrystals in in meso crystallization wells without the need for direct crystal harvesting. The requisite data quality for experimental phasing is achieved by accumulating diffraction signals from isomorphous crystals identified post-data collection. The method works in all experimental phasing scenarios and is particularly attractive with fragile, weakly diffracting microcrystals. The automated serial data collection approach can be readily adopted at most microfocus macromolecular crystallography beamlines.

## Abstract (web summary)

Huang et al. report a fully automated method for collecting and merging data from thousands of in meso-grown microcrystals. This in situ approach provides a fast and efficient way to determine the structures of membrane proteins from fragile microcrystals.

## Introduction

Membrane proteins perform essential roles in signal and energy transduction, metabolism, and transport and contribute to the structural integrity of cells. They account for close to a third of all cellular proteins and are important drug targets. High-resolution three-dimensional structural information is key to understanding how membrane protein work at a molecular level and can be used to inform structure-based drug design and discovery^1,2^. The vast majority of membrane protein structure work has been performed using macromolecular crystallography. Recent advances in cryogenic electron microscopy (cryo-EM) and de novo prediction methods will undoubtedly contribute to providing much-needed new structures^3,4^. However, many membrane proteins are still too small to be imaged at high resolution by cryo-EM and de novo prediction methods, as yet, do not provide atomic-level resolution. Macromolecular crystallography is limited too in that membrane protein crystals of high diffraction quality are difficult to generate. The lipid cubic phase (LCP) or in meso method of crystallization has made important contributions in this regard, in part, because proteins crystallize from within and into a native membrane-like environment^5^. In recent years, the in meso method accounts for close to 40% of all new unique membrane protein crystal structures (Supplementary Fig. 1).

Another challenge associated with membrane protein structure determination is that most new and interesting targets have novel structures with which the most common phasing method, molecular replacement, is rarely useful. In such cases, de novo structure determination by experimental phasing is required^6^. Of the 738 published unique membrane protein structures as of May 2018 (http://blanco.biomol.uci.edu/mpstruc/), 617 were solved by crystallography, of which 46% used experimental phasing with single-wavelength anomalous diffraction (SAD) the most popular de novo experimental phasing method (Supplementary Fig. 1). Experimental phasing comes with its own challenges. It requires highly accurate diffraction data, which are difficult to acquire especially when using small, fragile crystals of the type commonly encountered with membrane proteins. Heavy atom experimental phasing of membrane proteins with serial crystallography approaches has been demonstrated using crystals harvested with micro-meshes or standard loops^7–9^. But harvesting of the large numbers of crystals required for serial data collection is very time-consuming and particularly challenging for crystals grown in meso. This background serves to highlight the requirement for efficient and robust de novo phasing methods for use with membrane protein microcrystals.

**Fig. 1:**
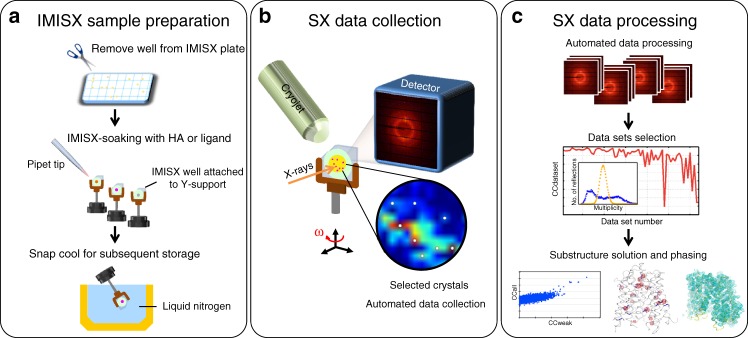
Overview of the IMISX-EP workflow for high-throughput in situ de novo phasing of in meso-grown microcrystals of membrane proteins. **a** IMISX crystallization, well preparation, and post-crystallization treatment, snap-cooling, and storage. **b** Automated grid scanning and serial data collection on hundreds of microcrystals in situ with a fast frame-rate detector. **c** Selection of isomorphous data sets based on intensity correlation coefficients (red line), analysis of crystal orientations (inset), and phase determination

**Fig. 2:**
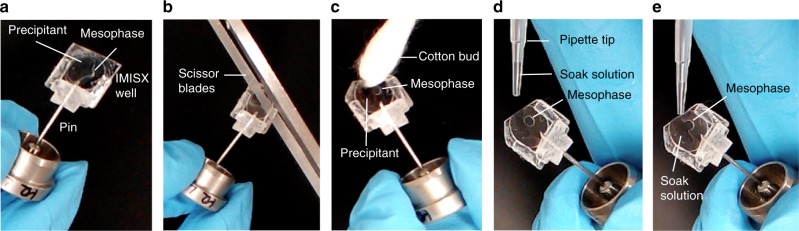
Steps involved in the heavy atom soaking of crystals growing in the lipid cubic mesophase inside IMISX wells for use in IMISX-EP. **a**, **b** Photographic images of the process of cutting open an IMISX well with a scissor, **c** wicking away excess precipitant solution with a cotton bud from around the crystal-laden mesophase, and **d**, **e** adding heavy atom containing precipitant solution by pipette

Responding to this critical need, we introduce here a fully automated method for collecting, selecting, and merging data from hundreds to thousands of in meso-grown microcrystals. This approach boosts the collective phasing signal of many tiny crystals to a level where de novo phasing of highly challenging membrane protein targets becomes not only possible but also fast and high-throughput. We refer to it as the in meso in situ serial crystallography—experimental phasing (IMISX-EP) method. IMISX-EP has three components: (1) in meso crystallization (and in situ soaking where appropriate), (2) fast, automated grid scanning, and serial data collection in situ, and (3) real-time data processing and selection of data sets followed by structure solution (Fig. 1). IMISX-EP eliminates one of the major bottlenecks subsequent to in meso crystallization—direct crystal harvesting, and enables in situ diffraction data collection by combining in meso crystallization and in situ serial synchrotron crystallography^10,11^ at cryogenic temperatures. In addition, the method provides convenient and highly effective in situ soaking capabilities (Fig. 2). We demonstrate the utility of the method with four real-life integral membrane proteins using all major anomalous phasing methods, and show that IMISX-EP provides a fast, efficient, and a direct means for de novo structure determination of membrane protein as microcrystals.

## Results

**Fig. 3:**
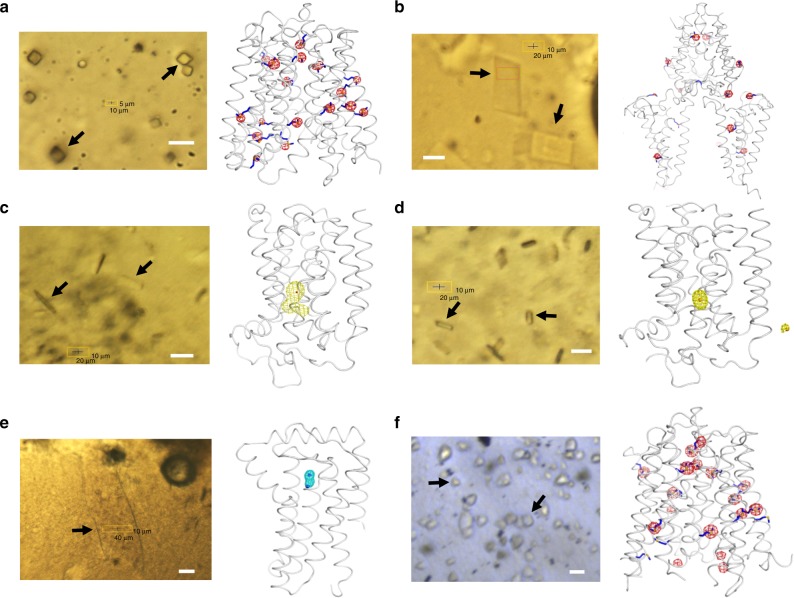
Structures of membrane proteins solved by IMISX-EP and the crystals used for data collection. Photographic images of crystals in IMISX wells held in the cryo-stream were recorded with an in-line microscope at beamline X06SA-PXI. **a** Se-PepT_St_, 2.7 Å, 89 crystals, two wells, Se-SAD, 18 Se. **b** Se/S-LspA, 3.0 Å, 497 crystals, 32 wells, Se-SAD, 12 Se. **c** Hg-BacA IMISX-soaking, 3.0 Å, 360 crystals, eight wells, Hg-SAD, 1 Hg. **d** Hg-BacA co-crystallization, 2.6 Å, 55 crystals, two wells, Hg-SAD, 2 Hg. **e** W-PgpB, 1.8 Å, one crystal, one well, W-SAD, 1 W. **f** S-PepT_St_, 2.7 Å, 1595 crystals, 59 wells, S-SAD, 13 S. Structures are shown in ribbon representation with anomalous sub-structures depicted as spheres and SHELXE anomalous Fourier maps contoured at 5 σ. The SHELXE anomalous Fourier map was calculated using the observed anomalous difference (|*F*_A_|) and the phases of the sub-structure determined using SHELXD. The black arrows point to representative crystals in each well. The white scale bar corresponds to 20 μm

The first demonstration of IMISX-EP was carried out using the most popular experimental phasing method, SAD phasing, with the Se-Met labeled proteins, PepT_St_ and LspA. Instead of harvesting mesophase-containing individual or clusters of crystals from each well by the traditional loop-harvesting method, the entire IMISX well was removed from the plate, mounted on a pin and snap-cooled in liquid nitrogen (Methods, Supplementary Fig. 2). This enabled in situ X-ray diffraction data collection to be carried out subsequently on all crystals in the IMISX well. From two such wells, 210 PepT_St_ crystals were measured, 145 partial data sets were indexed and processed, and 89 were selected on the basis of diffraction intensity correlation coefficient (*CC*_*dataset*_) (Supplementary Fig. 3a, Table 1 and Table 2) for successful Se-SAD phasing with SHELXD/E^12^ (Fig. 3a, Table 2, Supplementary Figs. 4a, 5a). The second example of Se-SAD phasing proved to be a particularly challenging case study for serial crystallography because the target protein, LspA, was incompletely Se-Met labelled. The average Se-Met incorporation was estimated at 59% based on the refined Se anomalous scattering signal (Methods). In this instance, the high-throughput feature of the IMISX-EP method proved invaluable enabling diffraction data to be collected from 974 LspA crystals in 32 IMISX wells (Table 1 and Table 2). The final data set derived from 497 crystals (Supplementary Fig. 3b). The substructure was determined by SHELXD and an interpretable map was generated by CRANK2^13^ (Fig. 3b, Table 2, Supplementary Figs. 4b, 5b).

**Table 1:** Data collection and refinement statistics*

	Se-PepT_St_ (6FMR)	Se/S-LspA (6FMS)	Hg-BacA soaking SAD (6FMT)	Hg-BacA soaking SIRAS	BacA Native SIRAS (6FMV)	Hg-BacA Co-crystallization (6FMW)	W-PgpB (6FMX)	S-PepT_St_ (6FMY)
*Data collection*								
Space group	*C*222_1_	*C*2	*C*222	*C*222	*C*222	*C*222	*I*222	*C*222_1_
Cell dimensions								
*a*, *b*, *c* (Å)	103.7, 110.9, 110.4	112.8, 110.1, 86.0	112.6, 145.6, 40.3	112.6, 145.6, 40.3	113.4, 144.4, 40.4	114.1, 145.1, 40.0	68.8, 76.8, 98.9	101.2, 110.2, 111.5
*α, β, γ* (°)	90, 90, 90	90, 97.1, 90	90, 90, 90	90, 90, 90	90, 90, 90	90, 90, 90	90, 90, 90	90, 90, 90
Wavelength (Å)	0.97854	0.97858	1.9	1.9	1.0	1.9	1.21371	2.0664
Resolution (Å)	49.54–2.70 (2.77–2.70)	46.27–3.00 (3.08–3.00)	44.58–3.00 (3.08–3.00)	44.56–3.00 (3.08–3.00)	40.39–2.30 (2.36–2.30)	44.45–2.60 (2.67–2.60)	35.6–1.79 (1.90–1.79)	49.29–2.70 (2.77–2.70)
*R*_meas_	0.44 (3.69)	0.32 (3.46)	0.48 (3.93)	0.38 (2.86)	0.50 (6.39)	0.38 (2.00)	0.10 (1.32)	0.32 (1.69)
*I* /σ *(I)*	8.90 (1.74)	9.14 (1.45)	10.62 (1.97)	10.34 (1.98)	9.85 (1.56)	8.95 (1.96)	7.93 (0.91)	40.2 (8.8)
*CC*_1/2_ (%)	99.4 (45.3)	99.6 (40.9)	99.7 (69.9)	99.8 (72.8)	99.5 (53.5)	99.4 (32.5)	99.7 (50.6)	100 (96.0)
Completeness (%)	100 (100)	99.9 (99.8)	100 (100)	100 (100)	100 (100)	99.9 (99.5)	91.7 (90.0)	100 (100)
Multiplicity	25.4 (23.8)	22.7 (22.7)	65.4 (60.7)	49.9 (47.2)	16.95 (14.09)	20.7 (15.7)	2.60 (2.50)	423.6 (378.1)
Refinement**								
Resolution (Å)	49.55–2.70	46.27–3.00	44.56–3.00	–	40.39–2.30	44.45–2.60	38.40–1.79	49.29–2.70
No. of unique reflections	33,746/1655	41,032/2058	12,841/641	–	15,224/761	19,671/975	39,988/2006	33,070/1661
*R*_work_/*R*_free_	0.23/0.25	0.23/0.27	0.24/0.28	–	0.19/0.22	0.22/0.25	0.24/0.26	0.21/0.25
No. of atoms								
Protein	3,649	4,990	1,896		2,081	2,061	1,587	3,448
Ligand/ion	244	466	89	–	197	57	129	320
Water	37	10	4	–	65	6	93	10
B-factor								
Proteins	53.7	80.4	60.4	–	38.9	51.5	33.3	47.4
Ligand/ion	62.6	87.4	69.1	–	58.4	60.3	51.5	61.3
Water	50.2	73.9	60.7	–	42.4	44.3	38.7	46.1
R.m.s. deviations								
Bond lengths (Å)	0.002	0.003	0.003	–	0.003	0.003	0.005	0.003
Bond angles (°)	0.443	0.525	0.528	–	0.611	0.640	0.822	0.705

*Data processing statistics are reported with *Friedel* pairs separated. Values in parentheses are for the highest resolution shell

**Refinement statistics are reported with *Friedel* pairs separated for all cases except BacA native SIRAS

**Table 2:** Sample consumption and phasing statistics

	Se-PepT_St_ (6FMR)	Se/S-LspA (6FMS)	Hg-BacA soaking SAD (6FMT)	Hg-BacA soaking SIRAS	BacA Native SIRAS (6FMV)	Hg-BacA Co-crystallization (6FMW)	W-PgpB (6FMX)	S-PepT_St_ (6FMY)
*Sample consumption*								
Heavy atom labeling	Se-Met	Se/S-Met	Hg-soaking	Hg-soaking	None	Hg-co-crystallization	W-co-crystallization	Native sulfur
Protein consumption (μg)	0.5	7.7	1.9	1.9	1.2	0.48	0.3	14.8
No. of wells	2	32	8	8	5	2	1	59
No. of crystals	210	974	968	968	125	66	1	6,639
No. of processed data sets	145	614	742	742	110	64	1	4,528
No. of merged data sets	89	497	360	271	94	55	1	1,595
Selection rate (%)	42	51	37	28	75	83	100	24
Total data (°)	1,335	4,970	3,600	2,710	940	1,170	140	15,950
*Phasing*								
Phasing method	Se-SAD	Se-SAD	Hg-SAD	Hg-SIRAS	Hg-SIRAS	Hg-SAD	W-SAD	S-SAD
SHELXD resolution range (Å)	49.54–3.20	44.35–4.20	44.58–3.30	44.56–3.30	–	44.45–4.00	35.60–1.80	49.29–3.50
SHELXD *CC*_*all*_/*CC*_*weak*_ (%)	44.8/21.6	41.5/16.5	29.4/17.1	23.3/16.8	–	38.5/24.0	39.1/20.1	31.0/12.6
Heavy atom sites	18 Se	12 Se	1 Hg	1 Hg	–	2 Hg	1 W	13 S

When Se-Met labeling compromises expression and/or protein and crystal quality, traditional heavy atom derivatization is an alternative and proven approach for experimental phasing. The heavy atom can be introduced either by co-crystallizing it with the protein or by soaking it into the crystal. We demonstrated the IMISX-EP approach for both heavy atom derivatization methods with two membrane proteins, BacA and PgpB. Indeed, BacA is the first de novo structure solved by the IMISX-EP method^14^. BacA crystals were derivatized by in situ soaking with HgCl_2_ in IMISX wells directly (Fig. 2). These wells provide an ideal environment in which to optimize heavy atom concentration and soaking time. Further, labeling benefits from the sticky, viscous, and nanoporous nature of the mesophase in which the crystals are suspended during soaking and buffer exchange (Methods, Fig. 2). From the 968 BacA crystals identified, two groups were created with which to evaluate two phasing methods (Supplementary Fig. 3c and 3d, Table 1 and Table 2). One contained 360, the other 271 data sets. The first group was used successfully for SAD phasing with SHELXD and CRANK2 (Fig. 3c, Supplementary Figs. 4c, 5c). The smaller, second group was combined with a high-resolution native data set for phasing with single isomorphous replacement with anomalous scattering (SIRAS), and the structure was phased readily with SHELXD/E (Table 2, Supplementary Figs. 4d, 5d). This example illustrates convincingly how SIRAS with a relatively weak heavy atom data set can be combined with a higher resolution native data set for experimental phasing in serial crystallography.

IMISX-EP using heavy atom co-crystallization was demonstrated with BacA and PgpB. BacA, was labeled with HgCl_2_ in solution ahead of setting up trials in IMISX crystallization plates. Of the 66 crystals measured from two IMISX wells, 64 were indexed and integrated, and 55 were selected (Supplementary Fig. 3e, Table 1 and Table 2). Mercury sites were identified with SHELXD, and an interpretable map was obtained by employing 25 cycles of SHELXE density modification with autobuilding (Fig. 3d, Table 2, Supplementary Figs. 4e, 5e). PgpB was pre-incubated with Na_2_WO_4_ and subsequently crystallized in the cubic mesophase. The crystal used for data collection was relatively large and measured 10 × 50 × 150 μm^3^. As the crystal had its flat face oriented approximately parallel to the windows of the well (Fig. 3e), it was possible to record 140° of data using a rotation angle of ± 70° from this one crystal without compromising data quality at high-tilt angles (Supplementary Fig. 6). Because of the strong anomalous signal from tungsten and the high diffraction resolution, the structure was solved with the relatively low redundancy of 2.6 and a completeness of 91% with SHELXD/E (Fig. 3e, Supplementary Figs. 4f, 5f). This example shows that the IMISX-EP approach can be used with large single crystals.

To demonstrate the full potential of IMISX-EP, the most-challenging experimental phasing method, native-SAD using the anomalous signal from light elements (Z ≤ 20) only, was attempted with unlabeled PepT_St_ crystals. The estimated *Bijvoet* ratio (*ΔF*/*F*) of sulfur (S)-PepT_St_ is ~ 1/100 (1%) at 6 keV (2.067 Å), which is approximately one-fourth that of Se-PepT_St_ at the Se absorption edge. Given the success of Se-SAD phasing using 89 Se-PepT_St_ crystals as described above, one expects that thousands of PepT_St_ microcrystals would be needed for native-SAD phasing. To expedite data collection on thousands of microcrystals, a high-throughput serial data collection protocol was implemented. It combined 50–100 Hz grid scanning of the entire 1–2 mm diameter LCP bolus with a micrometer-sized X-ray beam and automated data collection from each crystal identified on the basis of the grid scan (Methods, Supplementary Fig. 7). A total of 98,050° of data were recorded from 6639 crystals in 59 IMISX wells using 27 h of beamtime. Of these, 4528 crystals were indexed and processed and the final data derived from 1595 crystals included 15,950° of data (Supplementary Fig. 3f, g, Table 1 and Table 2). SHELXD located 13 of the 18 internal S sites in PepT_St_ and SHELXE produced an interpretable map (Fig. 3f, Table 2, Supplementary Figs. 4g, 5g).

## Discussion

It is clear from the case studies presented that IMISX-EP is a powerful and generally applicable high-throughput method. It combines the advantages of conventional crystallography with those of serial crystallography^7–11,15–19^ and transforms traditional micro-crystallography^20^ to an in situ serial crystallography method that is considerably more efficient and that requires miniscule quantities of protein. Specifically, in the most-challenging native-SAD phasing example above, < 20 μg PepT_St_ was used. Not only does the method extend phasing to microcrystals while minimizing the detrimental effects of radiation damage, it also effectively reduces both random and systematic errors in diffraction data to such an extent that the weak anomalous signal from light elements can be extracted reliably. At first blush, serial crystallography may appear to differ dramatically from conventional crystallography. In fact, the two are similar in that both seek to accumulate sufficient diffraction signal; by exploiting the voluminous diffraction of a large single crystal in conventional crystallography and by combining the diffraction volume of many microcrystals in serial crystallography^21,22^. Therefore, IMISX-EP very effectively improves the success rate of the phasing experiment by simple signal accumulation using a highly efficient serial crystallography method. It represents a paradigm shift from the conventional, subjective selection of crystals based on appearance, ease of harvesting, available harvesting time, and storage space to an exhaustive measurement of all crystals to the limits of radiation damage from which accurate data can be gleaned based upon a carefully chosen subset of isomorphous crystals.

In this study, the experimental phasing examples used for purposes of demonstration ranged from simple and easy to perform to extremely challenging. The strength of the phasing signal, as estimated by the *Bijvoet* ratio, ranges from 1% in the case of native-SAD PepT_St_ to 7% for tungsten phasing of PgpB. The observable anomalous peak height, which determines the success of a SAD experiment, is proportional to the square root of the number of observed reflections^23^, which in turn is related directly to the diffraction resolution. Therefore, experimental phasing becomes considerably more difficult in going from working with the high-resolution data available for heavy atom-labeled W-PgpB to the mid-resolution data available for native S-PepT_St_. This is clearly reflected in the total number of degrees of data used for successful phasing in six cases and is congruent with the results of comparable phasing studies in the literature, which include soluble proteins^7,9,11,17^ (Supplementary Fig. 8). The weaker the phasing signal, the more critical becomes the process of selecting out and combining isomorphous data sets. Different criteria and clustering methods have been tested and implemented recently^24–30^. For native-SAD PepT_St_ data that have a weak signal and a large number of data sets, an iterative procedure based on *CC*_*dataset*_ proved most effective. It rejects non-isomorphous data sets, whilst retaining weak but isomorphous data sets, which contribute to providing the needed anomalous signal.

The IMISX method takes advantage of the intrinsic viscosity of the cubic mesophase in which crystals grow. Thus, the entire crystal-laden bolus is contained in a confined space within the IMISX well, which can be cryogenically cooled for safe storage and to extend crystal lifetime in the X-ray beam. Another advantage is the ease with which heavy atom and ligand-soaking experiments can be performed in true in situ fashion without ever touching the crystals, which remain suspended in the viscous mesophase. Wider use of this convenient soaking feature will greatly facilitate mechanistic studies and drug discovery with membrane protein targets.

IMISX-EP has wide ranging applicability. It can be used with all experimental phasing approaches for membrane protein structure determination extending from traditional heavy atom derivatization and Se-Met labeling to the more recent native-SAD^6,21,31^ and emerging methods that include iodo-detergent labeling^32^ and fast halide soaking^9^. IMISX-EP is compatible with different plate and sample holder types^19,33–35^ and can be performed at most modern synchrotron macromolecular crystallography beamlines. It is likely to prove important in the effective utilization of next generation synchrotron sources such as the Diffraction Limited Storage Ring^36^. Furthermore, it is anticipated that the entire IMISX well mounting, crystal-laden bolus centering, rastering, and serial data collection process will be carried out unattended once the initial data collection parameters are known. Taken together, IMISX-EP, and serial crystallography in general, will contribute to having membrane protein structures included in the protein structure knowledge base to a level that reflects the frequency with which they are found in the cell. Given the importance of membrane proteins as therapeutic targets, these methods, in turn, will greatly facilitate high-throughput structure-based drug design and discovery.

## Methods

### Protein purification

Four proteins were used in this study as follows: the peptide transporter, PepT_St_, from *Streptococcus thermophilus*^37^, the lipoprotein signal peptidase II, LspA, from *Pseudomonas aeruginosa* (PAO1)^38^, the undecaprenyl-pyrophosphate phosphatase, BacA, from *Escherichia coli* (K-12)^39^, and the phosphatidic acid phosphatase, PgpB, from *Bacillus subtilis*^40^. Se-LspA, PgpB, BacA, and native S-PepT_St_ were produced recombinantly and purified from biomass following published protocols^37–40^. Se-PepT_St_ was produced by using *E. coli* C43 (DE3) (NEB) cells with the pWaldo-*dtpT* plasmid. Cells were grown in SelenoMet™ Medium (Molecular Dimensions) supplemented with 50 mg/L Se-Met (Sigma) and 50 mg/L kanamycin (Melford) following supplier’s instructions. Se-PepT_St_ was purified by following a published protocol^37^. All protein samples were stored at − 80 °C.

### Crystallization

Crystals were grown from purified concentrated proteins by the LCP method^41^ in IMISX plates^10^. Complexes with globomycin (Sigma), cefadroxil (Sigma), and Ala-Phe (H-Ala-Phe-OH) (BACHEM) were used in the crystallization of Se-LspA, Se-PepT_St_, and native S-PepT_St_, respectively. Co-crystallization of Hg-derivatized BacA and W-derivatized PgpB was performed by pre-incubating protein solution at 4 °C with 2 mM HgCl_2_ (Hampton) for 10 min and 100 mM Na_2_WO_4_ (Hampton) for 30 min, respectively, before setting up crystallization trials. For Se-Met substituted LspA, Hg-derivatized BacA and W-derivatized PgpB, the mesophase was produced by homogenizing two volumes of protein solution at ~ 12 mg/mL with three volumes of monoolein (9.9 monoacylglycerol, MAG) (NuCheck) in a LCP coupled syringe mixing device at 20 °C^42^. A similar protocol was used with PepT_St_ with the following modifications. The hosting lipid, 7.8 MAG (Avanti), was used for native S-PepT_St_ and Se-PepT_St_ using equal volumes of lipid and protein solution to make the mesophase, and the concentration of the protein solution was 10 mg/mL. Crystallization trials were set up robotically at 20 °C using 50 nL protein-laden mesophase and 800–1000 nL precipitant solution for Se-PepT_St_, Se-LspA, Hg-derivatized BacA, and W-derivatized PgpB. In the case of native S-PepT_St_, crystals were grown in syringes. For this purpose, 20 μL protein-laden LCP was injected into a 500 μL-syringe containing 400 μL precipitant solution^43^. Proteins were crystallized under the following conditions: Se-PepT_St_ (21–22 %(v/v) PEG- 400, 250–325 mM ammonium dihydrogen phosphate, 100 mM HEPES, pH 7.0, and 10 mM cefadroxil), Se-LspA (35–43 %(v/v) PEG-400, 100 mM MES, pH 5.6–6.0, and 60–100 mM ammonium phosphate monobasic), Hg-derivatized BacA (40 %(v/v) PEG-400, 300–500 mM ammonium citrate dibasic and 100 mM sodium citrate pH 5.0), W-derivatized PgpB (40 %(v/v) PEG-400, 100 mM HEPES pH 7 and 100 mM lithium citrate tribasic tetrahydrate) and native S-PepT_St_ (21–22 %(v/v) PEG-400, 250–325 mM ammonium dihydrogen phosphate, 100 mM HEPES, pH 7.0, and 10 mM Ala-Phe). Crystals of native-BacA for soaking experiments were grown in the same precipitant as was used for Hg-derivatized BacA crystal production. Se-PepT_St_ crystals grew as pyramids with an average size of 5 × 10 × 10 μm^3^. Se-LspA crystals grew as thin plates with an average size of 10 × 20 × 50 μm^3^. BacA crystals grew as thin plates with an average size of 5 × 10 × 20 μm^3^. Hg-BacA crystals grew as thin plates with an average size of 3 × 5 × 15 μm^3^. W-PgpB crystal grew as plates to a size of 10 × 50 × 150 μm^3^. Native S-PepT_St_ crystals grew as pyramids with an average size of 10 × 10 × 10 μm^3^.

### IMISX-soaking and sample preparation

All of the samples used for data collection were prepared using the IMISX method as described previously^10,11^ with two important modifications. First, a Y-shaped holder (Supplementary Fig. 2) was used to secure the flexible IMISX well stably in the cryo-stream for effective raster scanning and data collection (Supplementary Fig. 7). Second, soaking crystals with heavy atoms was performed directly in the IMISX well without touching the hosting mesophase or the crystals therein. For this purpose, the intact well containing the crystal-laden mesophase bolus was removed from the double-sandwich IMISX plate and was mounted on a Y-support. One corner of the well was snipped off with a scissor and precipitant solution was wicked away from around the mesophase bolus using a cotton bud or tissue paper. Heavy atom reagent, dissolved in the same precipitant solution, was pipetted into the well via the open corner and manipulated so as to make contact with and to fully bathe the mesophase bolus. After a period of incubation at 20 °C (Rumed incubator, model 3101), the IMISX well was snap-cooled immediately in liquid nitrogen (Fig. 1). Samples were placed in pucks and shipped in a Dewar to the Swiss Light Source for measurement. Screening for optimum heavy atom labeling conditions was performed directly in IMISX wells in a process that involved different soaking times, heavy atom concentrations and heavy atom types chosen based on BacA sequence analysis in conjunction with the HATODAS II server^44^. Soaking with 10 mM HgCl_2_ for 70 min provided sufficient labeling for successful experimental phasing.

### Automated serial data collection

X-ray diffraction experiments were carried out on protein crystallography beamlines X06SA-PXI and X10SA-PXII at the Swiss Light Source, Villigen, Switzerland. Data were collected at 100 K using cryo-cooled IMISX wells in a cryo-stream, as described^11^. Measurements were made in steps of 0.1–0.2° at a speed of 0.1 s/step with either an EIGER 16 M or a PILATUS 6M-F detector operated in continuous/shutterless data collection mode^45^. Beam size was adjusted according to crystal dimensions to optimize signal/noise for data collection and typically measured 10 × 10 μm^2^ or 20 × 20 μm^2^. With the Swiss Light Source data acquisition software (DA+)^46^ an automated serial data collection protocol (CY+) was developed enabling grid scan rates of 50–100 Hz^47^. This made it possible to raster scan an entire 2 × 2 mm^2^ mesophase bolus with X-rays at micrometer resolution in about 8 min. The resulting finely sampled grid map accurately located all crystals in the bolus and provided a ranking of diffraction quality in heat map form. Immediately after completing the grid scan, the automated data collection protocol CY+ was launched for serial data collection on crystals above a defined diffraction quality threshold over a specified rotation range (typically 10–20°) and a defined beam attenuation. Following this protocol, a few hundred crystals were measured in an hour (Supplementary Fig. 7). Radiation damage per crystal was maintained below 5 MGy for all proteins except Se-PepT_St_ where a dose of 17 MGy per crystal was used.

### Data processing, selection, and merging

In serial crystallography, real-time data analysis is indispensable. This was implemented in the current IMISX-EP workflow by combining parallel data processing using XDS with data set selection and merging based on correlation coefficient and diffraction signal strength (Supplementary Fig. 3). Data collection wedges of 10–140° per crystal were indexed and processed using XDS^48,49^, as described^11^. For data set selection, all data sets were initially sorted in ascending order based on the averaged *R*_*meas*_ values calculated from the three lowest resolution shells of each data set. Next, the sorted data sets were scaled and merged with XSCALE to obtain a preliminary scaled and merged data set. Then, the correlation coefficients (*CC*_dataset_) based on intensities in resolution shells were calculated between the preliminary merged data and each individual data set. The selection of data sets was carried out on the basis of the *CC*_dataset_ values of each data set in a chosen resolution shell and the data sets below a certain *CC*_dataset_ were rejected. This *CC*_dataset_ selection process is usually carried out in an iterative manner based on a case-by-case basis. With Se-LspA, an additional selection based on the asymptotic < *I*/σ > ratio (ISa)^50^, as determined by XSCALE, was used to remove outlier data sets before *CC*_dataset_ selection. Final data sets were scaled and merged with XSCALE. Data collection and processing statistics are provided in Table 1.

### Structure determination and refinement

The SAD method was employed for experimental phasing using anomalous diffraction data sets from crystals of Se-PepT_St_, Se/S-LspA, W-PgpB, Hg-BacA (soaking), Hg-BacA (co-crystallization), and native S-PepT_St_. The SIRAS method was also used for Hg-BacA (soaking). Heavy atom location, structure phasing, and density modification were performed using the HKL2MAP^51^ interface of SHELXC/D/E^12^ for all structures and produced interpretable electron density maps for Se-PepT_St_, Hg-BacA (soaking, SIRAS), Hg-BacA (co-crystallization), W-PgpB, and native S-PepT_St_. Additional phase improvement and iterative auto-model building were carried out with CRANK2^13^ for Se/S-LspA and Hg-BacA (soaking, SAD). From the experimentally phased maps, BUCCANEER^52^ and PHENIX AutoBuild^53^ were used for initial model building and models were completed manually using COOT^54^. Phenix.refine^55^ and BUSTER^56^ were used during the refinement of all structures. Phasing and refinement statistics are reported in Table 1 and Table 2. Figures of molecular structures were generated with PyMOL^57^.

### Refinement of anomalous scattering factor *f”*

The anomalous scattering factor (*f”*) of Se in the Se-Met of PepT_St_ and LspA was refined using phenix.refine^55^ as follows. First, the atomic coordinates and B-factors were refined with standard PHENIX refinement to converged *R*_*work*_/*R*_*free*_ values. Then, the B-factor of each Se atom was set to the average B-factor of its neighboring carbon atoms (Cγ and Cε) and its occupancy was set to one. Finally, the anomalous scattering factor *f”*, the atomic scattering factor *f’*, and atomic coordinates were refined using fixed B-factor and occupancy values for all Se atoms. The refined average values of *f”* were 5.38 and 3.16 for PepT_St_ and LspA, respectively. This result indicates a Se-Met substitution value of ~ 59% in LspA using PepT_St_ as reference with 100% substitution.

### Code availability

Custom computer codes for correlation coefficient based data set selection and diffraction data are available at https://github.com/ranganawarshamanage/mxt.

### Data availability

All diffraction data and refined models have been deposited in the Protein Data Bank under PDB identifiers 6FMR (Se-PepT_St_), 6FMS (Se/S-LspA), 6FMT (Hg-BacA soaking SAD), 6FMV (BacA Native SIRAS), 6FMW (Hg-BacA co-crystallization), 6FMX (W-PgpB), and 6FMY (PepT_St_). All data sets generated during the current study are available upon request.

## Electronic supplementary material

Supplementary information
