# Supplementary material for: In situ serial crystallography for rapid de novo membrane protein structure determination
Source: Commun Biol. 2018 Aug 27;1:124. doi: 10.1038/s42003-018-0123-6 (PMC6123769; doi:10.1038/s42003-018-0123-6)
Supplement: Supplementary file 1 — Supplementary information [file 42003_2018_123_MOESM1_ESM.pdf]

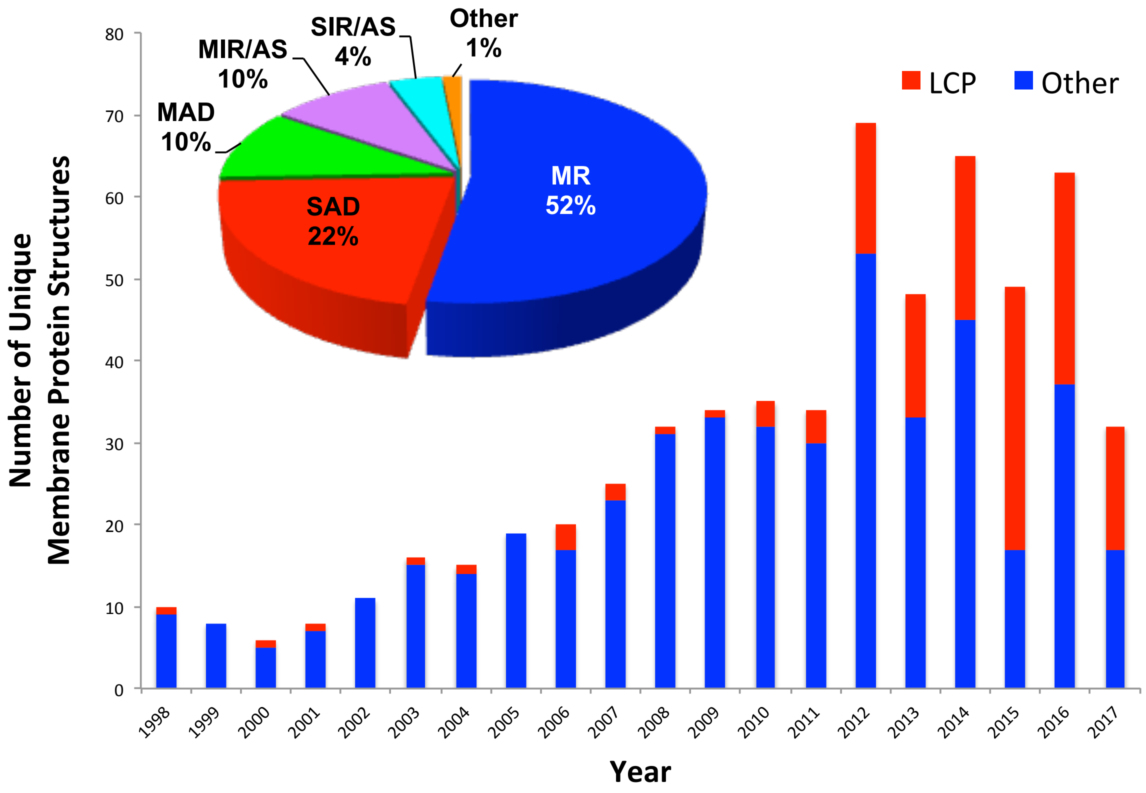

Supplementary Figure 1. Annual number of PDB records for unique membrane protein structures solved using crystals grown by the LCP and other methods. The inset shows the distribution by phasing method for all unique membrane protein crystal structures in the PDB.

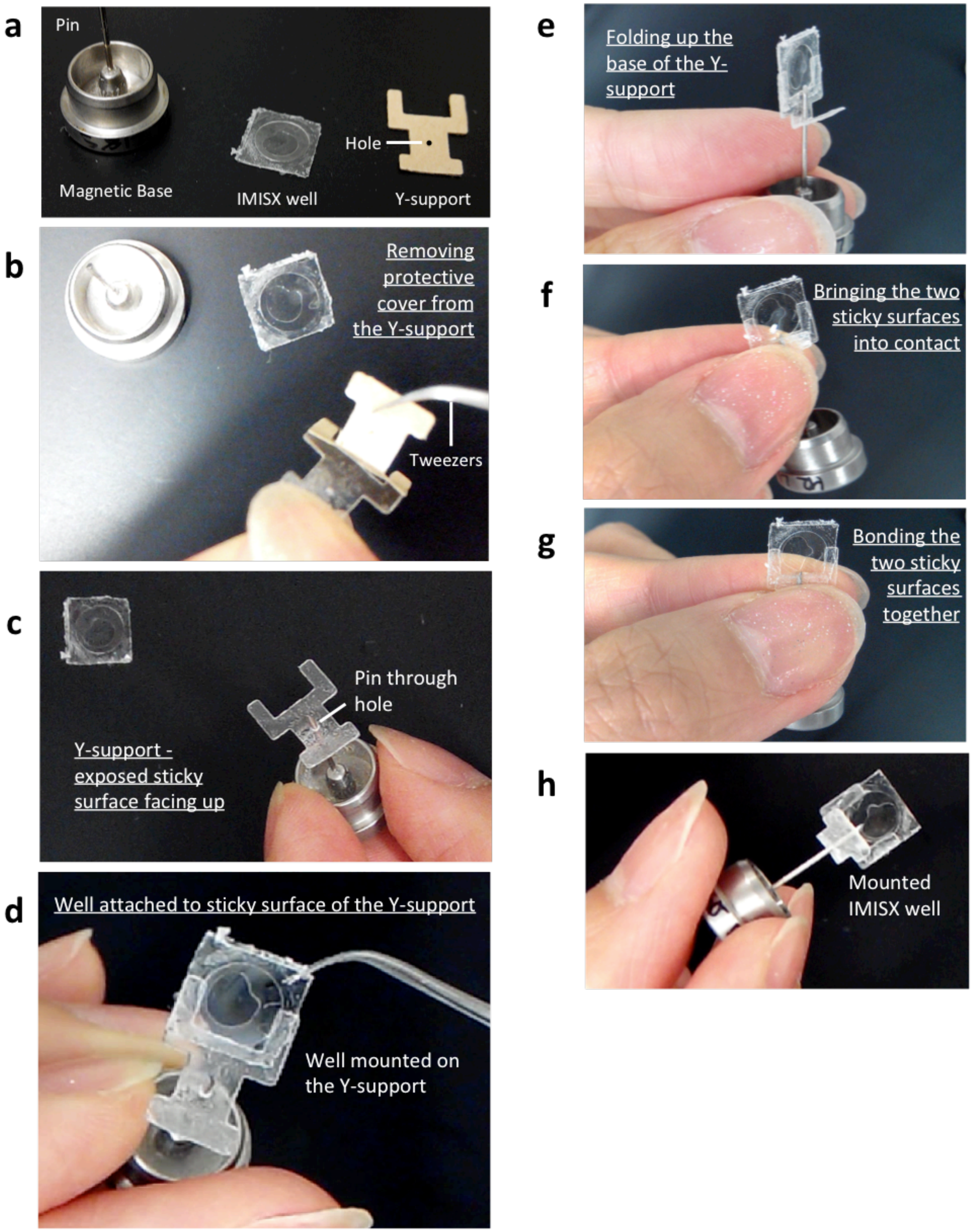
a
Pin
Magnetic Base
IMISX well
Hole
Y-support
b
Removing protective cover from the Y-support
Tweezers
c
Pin through hole
Y-support - exposed sticky surface facing up
d
Well attached to sticky surface of the Y-support
Well mounted on the Y-support
e
Folding up the base of the Y-support
f
Bringing the two sticky surfaces into contact
g
Bonding the two sticky surfaces together
h
Mounted IMISX well

Supplementary Figure 2. Steps involved in securing an IMISX well to a Y-shaped support and subsequently to a mounting pin. (**a**) Parts: mounting pin in base, IMISX well, Y-support with a centering hole and made from double-stick tape. (**b**) Protective cover is removed from one face of the Y-support. (**c**) The pin is passed through the centering hole in the Y-support sticky face up. (**d**) The well is positioned on the exposed sticky surface of the Y-support. The precipitant that surrounds the crystal-laden mesophase is seen clearly in the IMISX well. (**e**) (**f**) and (**g**) The Y-support is folded back on itself and the two parts pressed together to secure the well firmly to the pin. (**h**) The IMISX well securely attached to the pin on a base ready for precipitant removal, heavy atom or ligand soaking and/or snap cooling in liquid nitrogen (**Fig. 1 and Fig. 2**).

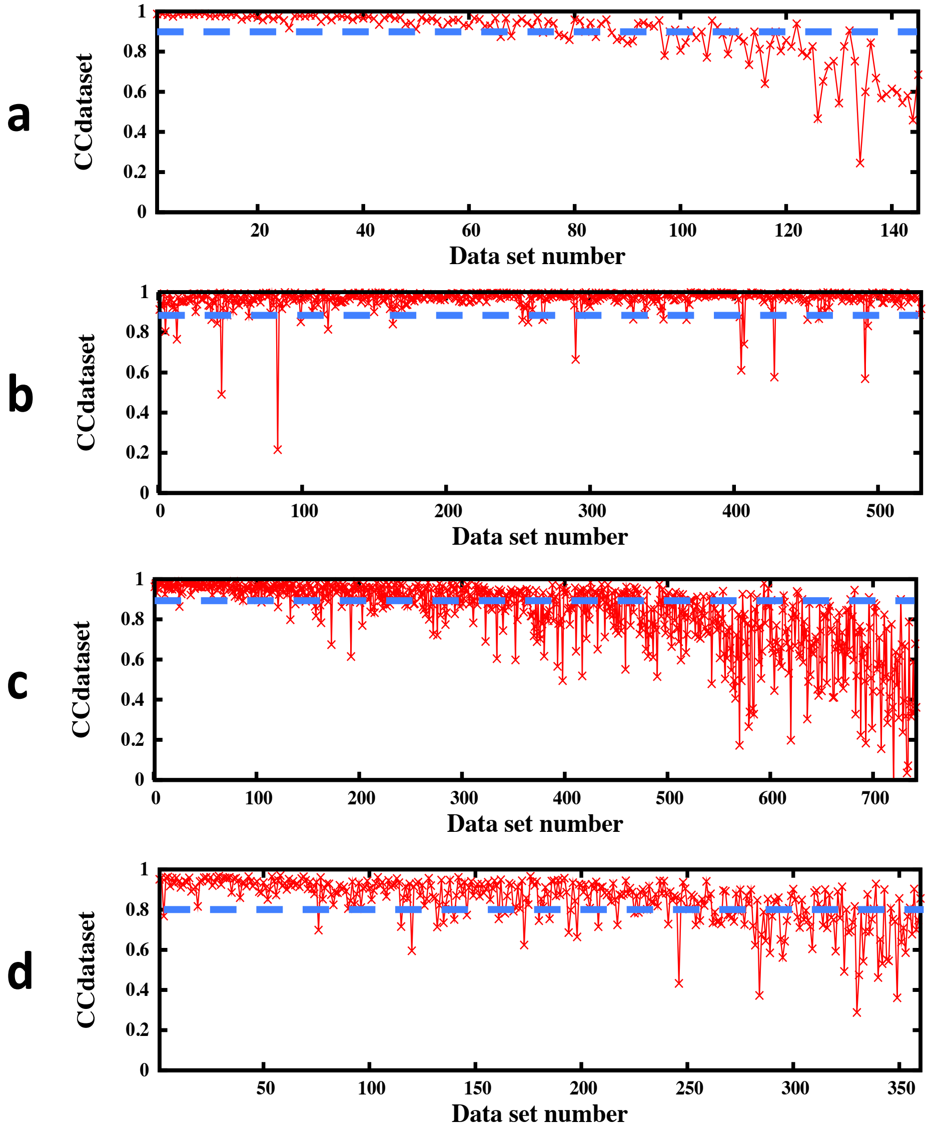
a
CCdataset
Data set number
b
CCdataset
Data set number
c
CCdataset
Data set number
d
CCdataset
Data set number

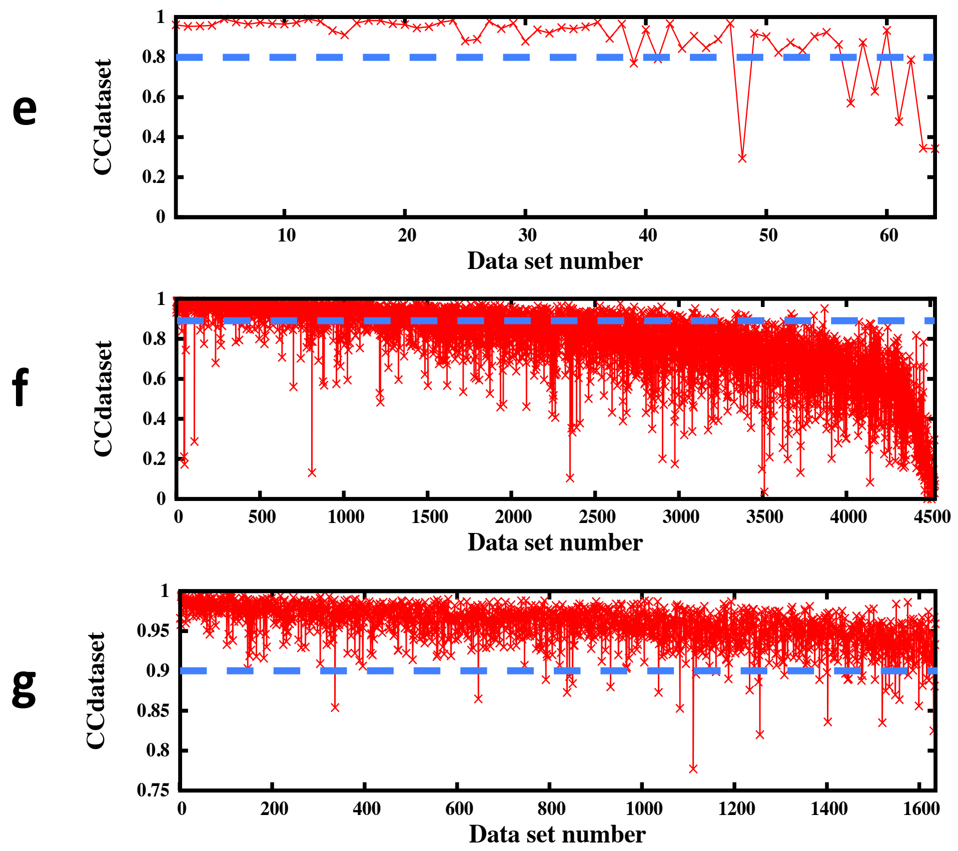

Supplementary Figure 3. Intensity correlation coefficient ($CC_{dataset}$) based data set selection. (**a**) Se-PepT$_{St}$. Of 145 data sets, 89 were selected that had $CC_{dataset}$ > 0.9 in the resolution range 4.88-4.23 Å. (**b**) Se-LspA. Of 530 data sets (from an ISa selection from 614 data sets), 497 were selected that had $CC_{dataset}$ > 0.9 in the resolution range 46.27-3.0 Å. (**c**) Hg-BacA IMISX-soaking-SAD. Of 742 data sets, 360 were selected that had $CC_{dataset}$ > 0.9 in the resolution range 6.65-5.45 Å. (**d**) Hg-BacA IMISX-soaking-SIRAS. Of 360 data sets, 271 were selected that had $CC_{dataset}$ > 0.8 in the resolution range 4.73-4.23 Å. (**e**) Hg-BacA co-crystallization. Of 64 data sets, 55 were collected that had $CC_{dataset}$ > 0.8 in the resolution range 4.10-3.67 Å. (**f**) Native S-PepT$_{St}$. Of 4,528 data sets, 1,635 were selected that had $CC_{dataset}$ > 0.9 in the resolution range 4.26-3.81 Å. (**g**) Native S-PepT$_{St}$. Of the 1,635 data sets, 1,595 were selected that had $CC_{dataset}$ > 0.9 in the resolution range 4.91-4.26 Å.

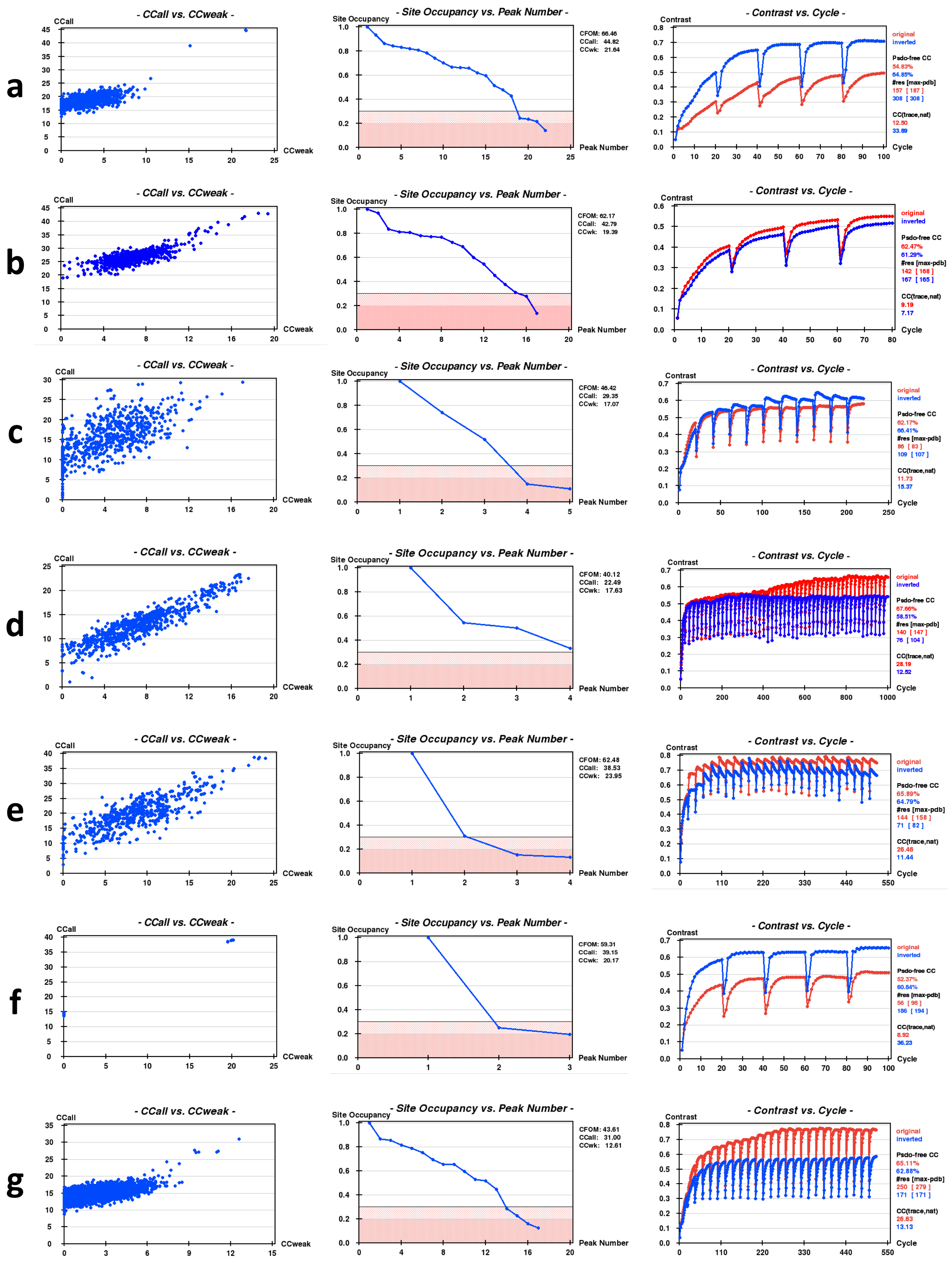
a
b
c
d
e
f
g
- CCall vs. CCweak -
CCall
CCweak
- Site Occupancy vs. Peak Number -
Site Occupancy
Peak Number
- Contrast vs. Cycle -
Contrast
Cycle
CFOM: 66.46
CCall: 44.82
CCwk: 21.64
original
inverted
Psdo-free CC
54.83%
64.85%
#res [max-pdb]
157 [187]
308 [308]
CC(trace,nat)
12.50
33.89
CFOM: 62.17
CCall: 42.79
CCwk: 19.39
original
inverted
Psdo-free CC
62.47%
61.29%
#res [max-pdb]
142 [168]
167 [165]
CC(trace,nat)
9.19
7.17
CFOM: 46.42
CCall: 29.35
CCwk: 17.07
original
inverted
Psdo-free CC
62.17%
66.41%
#res [max-pdb]
86 [83]
109 [107]
CC(trace,nat)
11.73
15.37
CFOM: 40.12
CCall: 22.49
CCwk: 17.63
original
inverted
Psdo-free CC
67.66%
58.51%
#res [max-pdb]
140 [147]
76 [104]
CC(trace,nat)
28.19
12.52
CFOM: 62.48
CCall: 38.53
CCwk: 23.95
original
inverted
Psdo-free CC
65.89%
64.79%
#res [max-pdb]
144 [158]
71 [82]
CC(trace,nat)
26.46
11.44
CFOM: 59.31
CCall: 39.15
CCwk: 20.17
original
inverted
Psdo-free CC
52.37%
60.84%
#res [max-pdb]
56 [98]
186 [194]
CC(trace,nat)
8.92
36.23
CFOM: 43.61
CCall: 31.00
CCwk: 12.61
original
inverted
Psdo-free CC
65.11%
62.88%
#res [max-pdb]
250 [279]
171 [171]
CC(trace,nat)
26.63
13.13

Supplementary Figure 4. Sub-structure determination and phasing with SHLEXC/D/E. (Left) SHELXD correlation coefficients (CC_{all}/CC_{weak}) between observed and calculated anomalous differences. (Middle) Site occupancies of anomalous sub-structures. A major site usually has an occupancy above 0.3. (Right) Hand-determination from phased electron density maps. The red and blue curves represent the two hands; the one with the higher contrast is the correct hand. (**a**) Se-PepT$_{St}$. (**b**) Se-LspA. (**c**) Hg-BacA IMISX-soaking-SAD. (**d**) Hg-BacA IMISX-soaking-SIRAS. (**e**) Hg-BacA co-crystallization. (**f**) W-PgpB. (**g**) Native S-PepT$_{St}$.

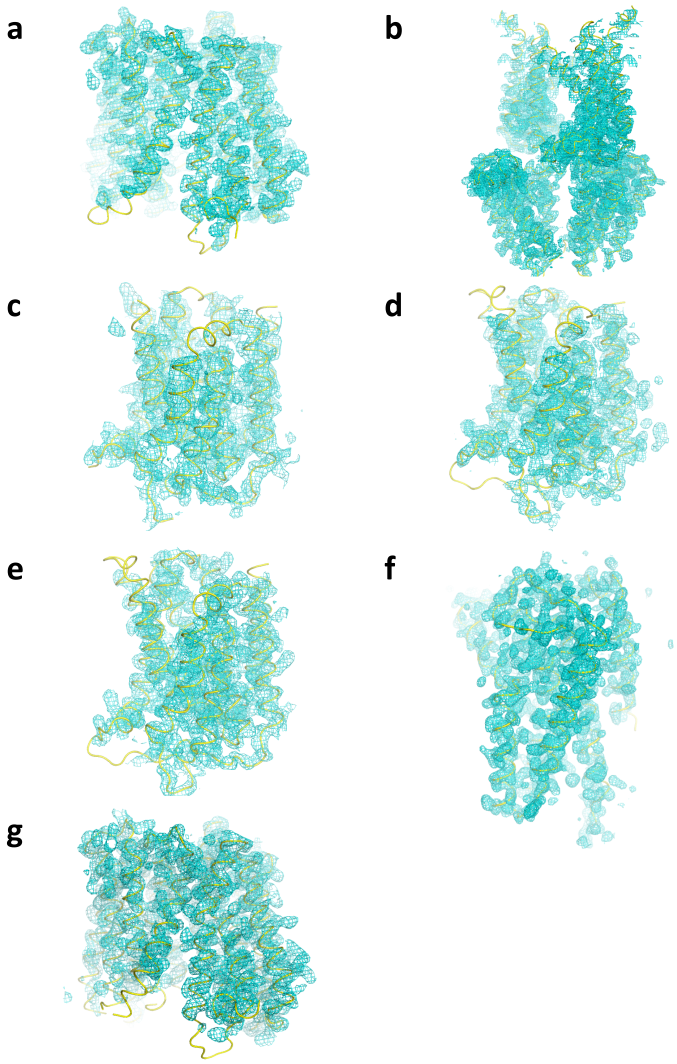

Supplementary Figure 5. Experimentally phased electron density maps contoured at 1 σ (green mesh) with C_α traces (yellow) of the final refined models superimposed. (**a**) Se-PepT$_{St}$. (**b**) Se/S-LspA. (**c**) Hg-BacA IMISX-soaking-SAD. (**d**) Hg-BacA IMISX-soaking-SIRAS. (**e**) Hg-BacA co-crystallization. (**f**) W-PgpB. (**g**) Native S-PepT$_{St}$. Maps in (a), (d), (e), (f) and (g) were calculated with SHELXE using the observed structure factors and the phases and FOM values obtained after density modification and polyalanine chain-tracing. Maps in (b) and (c) are weighted 2Fo-Fc maps from REFMAC5[1] after density modification, automatic model building and refinement using CRANK2.

a

b

c

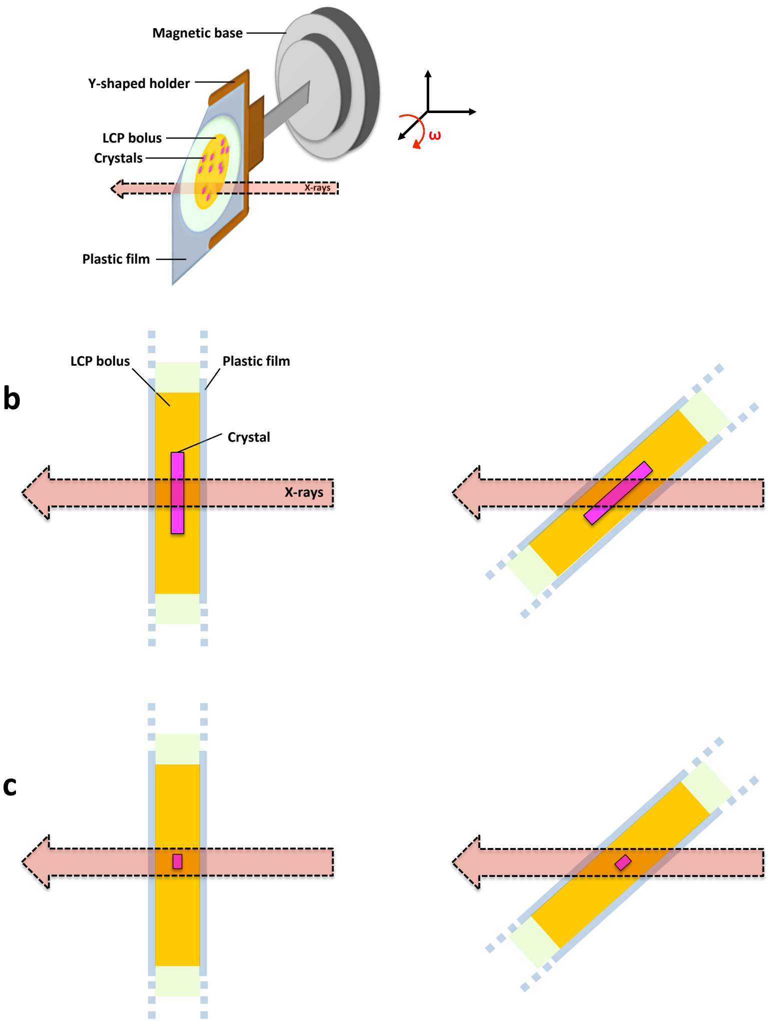

Supplementary Figure 6. Illustration of the diffraction geometry in an IMISX well. (**a**) Overview of the sample setup. (**b**) Both the diffraction volume of the crystal (magenta) and the volume of the bathing mesophase (orange) and COC films (blue, plastic films) intercepted by the X-ray beam (pink) increase with tilt angle (left to right panel) when the size of the beam is less than that of the crystal. (**c**) The diffraction volume of the crystal remains unchanged while the volume of the bathing mesophase and COC films intercepted by the X-ray beam increases with tilt angle (left to right panel) when the size of the beam is larger than that of the crystal.

a

b

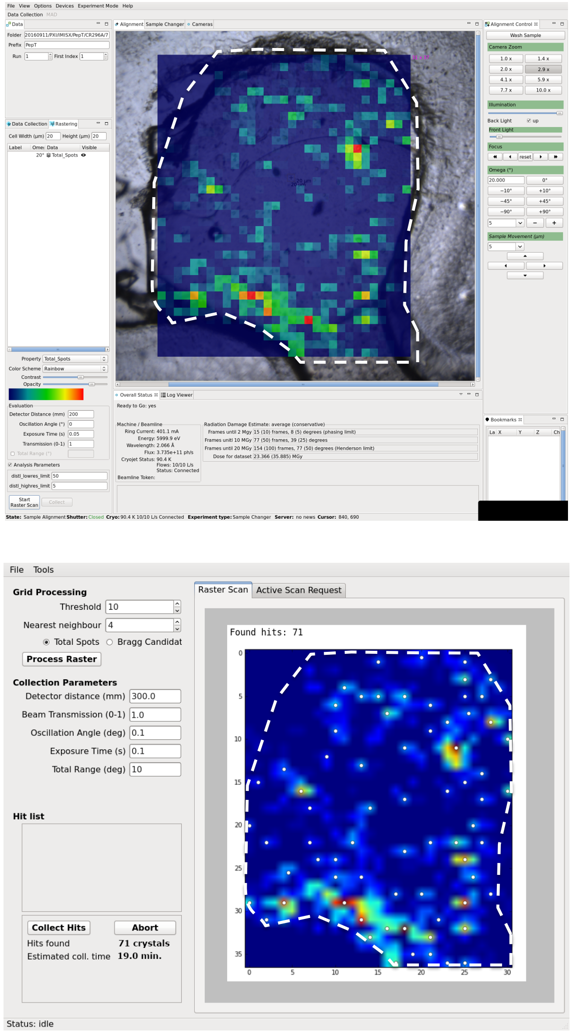

Supplementary Figure 7. Screenshots of the fast grid scan GUI (DA+) and automated serial data collection GUI (CY+). (**a**) DA+ GUI: A grid scan covers the complete LCP bolus (outlined with a dashed line) with the diffraction hits represented as a heatmap. The grid scan parameters are configured in the lower-left panel of the GUI. (**b**) CY+ GUI: The selected crystal hits, identified by DA+ in (a), are labelled automatically with white dots and overlaid on the DA+ grid scan heatmap. The outline of the LCP bolus is shown so that panels (a) and (b) can be compared. The crystal hits selection criteria and parameters for serial data collection are configured in the left panel of the GUI.

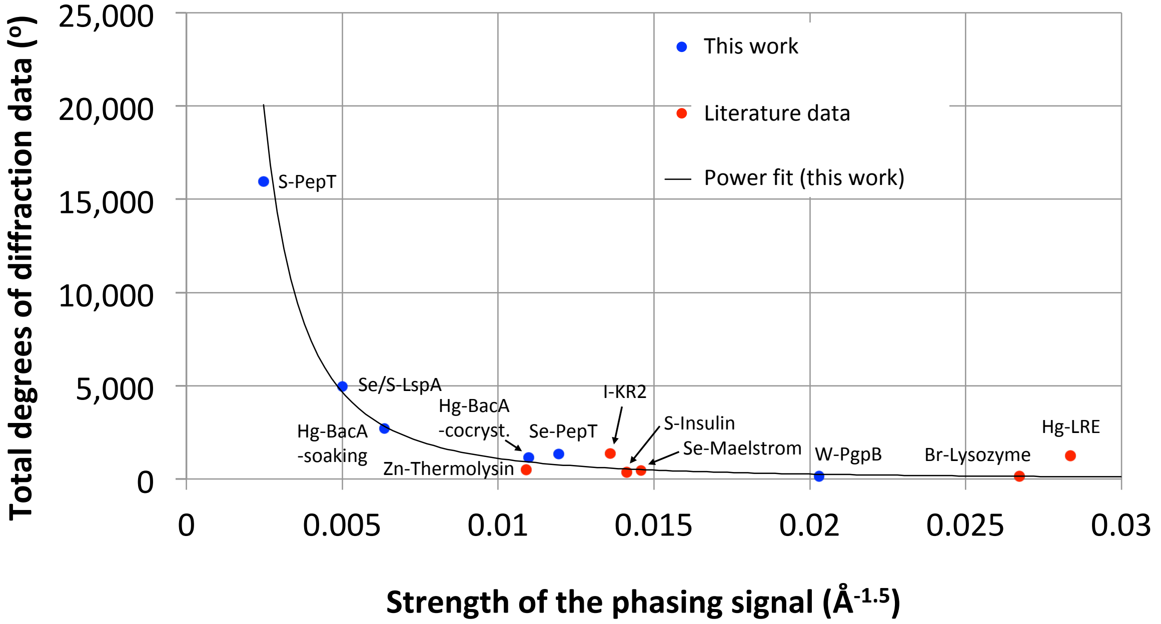

Supplementary Figure 8. Dependence of the total number of degrees of diffraction data required for experimental phasing by SAD on the strength of the phasing signal as observed in this study and in comparable work. The ordinate shows the total number of degrees of data used to solve structures of W-PgbB (140°), Se-PepT$_{St}$ (1,335°), Hg-BacA co-crystallization (1,170°), Hg-BacA soaking (3,600°), Se/S-LspA (4,970°), and native S-PepT$_{St}$ (15,950°) (this work, blue data points) and of S-Insulin[1] (360°), Br-Lysozyme[2] (140°), Zn-Thermolysin[3] (490°), Se-Maelstrom[3] (450°), Hg-LRE[4] (1,237°) and I-KR2[5] (1,360°) (literature, red data points). The abscissa shows the phasing signal strength calculated as the product of the *Bijvoet* ratio ($<|\Delta F^{\pm}|> / <|F|>$) and $(1/d)^{1.5}$ where *d* is the measured diffraction resolution. The data from this work were fitted with a power series ($Y = 0.0767 X^{-2.08}$, $R^2=0.938$) to guide the eye.

Supplementary References

1. Murshudov, G.N. et al. REFMAC5 for the refinement of macromolecular crystal structures. *Acta Crystallogr D Biol Crystallogr* **67**, 355-67 (2011).
2. Huang, C. Y. *et al.* In meso in situ serial X-ray crystallography of soluble and membrane proteins at cryogenic temperatures. *Acta Crystallogr D Struct Biol* **72,** 93–112 (2016).
3. Zander, U. *et al.* MeshAndCollect: an automated multi-crystal data-collection workflow for synchrotron macromolecular crystallography beamlines. *Acta Crystallogr. D Biol. Crystallogr.* **71,** 2328–2343 (2015).
4. Hasegawa, K. *et al.* Development of a dose-limiting data collection strategy for serial synchrotron rotation crystallography. *J. Synchrotron Radiat.* **24,** 29–41 (2017).
5. Melnikov, I. *et al.* Fast iodide-SAD phasing for high-throughput membrane protein structure determination. *Sci Adv* **3,** e1602952 (2017).
